# Supplementary material for: Profile of individuals served and presumed coverage of Psychosocial Care Centers (CAPS) in Brazil: A study of the period 2013–2019
Source: PLoS One. 2024 Sep 6;19(9):e0308274. doi: 10.1371/journal.pone.0308274 (PMC11379171; doi:10.1371/journal.pone.0308274)
Supplement: S1 Table — (DOCX) [file pone.0308274.s001.docx]

| Table S1 – Calculation memory for presumed coverage of CAPS by states and Brazil in 2013. | | | | | | | | | |
| --- | --- | --- | --- | --- | --- | --- | --- | --- | --- |
| **STATE** | **CAPS I** | **CAPS I REF** | **CAPS I POP** | **CAPS II** | **CAPS II REF** | **CAPS II POP** | **CAPS III** | **CAPS III REF** | **CAPS III POP** |
| AC | 0 | 50,000 | 0 | 1 | 100,000 | 100000 | 0 | 150,000 | 0 |
| AL | 45 | 50,000 | 2250000 | 6 | 100,000 | 600000 | 0 | 150,000 | 0 |
| AM | 13 | 50,000 | 650000 | 4 | 100,000 | 400000 | 1 | 150,000 | 150000 |
| AP | 0 | 50,000 | 0 | 0 | 100,000 | 0 | 0 | 150,000 | 0 |
| BA | 139 | 50,000 | 6950000 | 37 | 100,000 | 3700000 | 3 | 150,000 | 450000 |
| CE | 54 | 50,000 | 2700000 | 35 | 100,000 | 3500000 | 7 | 150,000 | 1050000 |
| DF | 1 | 50,000 | 50000 | 3 | 100,000 | 300000 | 0 | 150,000 | 0 |
| ES | 9 | 50,000 | 450000 | 7 | 100,000 | 700000 | 0 | 150,000 | 0 |
| GO | 26 | 50,000 | 1300000 | 13 | 100,000 | 1300000 | 2 | 150,000 | 300000 |
| MA | 40 | 50,000 | 2000000 | 15 | 100,000 | 1500000 | 2 | 150,000 | 300000 |
| MG | 98 | 50,000 | 4900000 | 46 | 100,000 | 4600000 | 11 | 150,000 | 1650000 |
| MS | 11 | 50,000 | 550000 | 5 | 100,000 | 500000 | 1 | 150,000 | 150000 |
| MT | 26 | 50,000 | 1300000 | 3 | 100,000 | 300000 | 0 | 150,000 | 0 |
| PA | 37 | 50,000 | 1850000 | 16 | 100,000 | 1600000 | 2 | 150,000 | 300000 |
| PB | 44 | 50,000 | 2200000 | 7 | 100,000 | 700000 | 5 | 150,000 | 750000 |
| PE | 38 | 50,000 | 1900000 | 22 | 100,000 | 2200000 | 4 | 150,000 | 600000 |
| PI | 36 | 50,000 | 1800000 | 7 | 100,000 | 700000 | 1 | 150,000 | 150000 |
| PR | 42 | 50,000 | 2100000 | 23 | 100,000 | 2300000 | 3 | 150,000 | 450000 |
| RJ | 36 | 50,000 | 1800000 | 44 | 100,000 | 4400000 | 3 | 150,000 | 450000 |
| RN | 16 | 50,000 | 800000 | 10 | 100,000 | 1000000 | 1 | 150,000 | 150000 |
| RO | 13 | 50,000 | 650000 | 5 | 100,000 | 500000 | 0 | 150,000 | 0 |
| RR | 5 | 50,000 | 250000 | 1 | 100,000 | 100000 | 1 | 150,000 | 150000 |
| RS | 71 | 50,000 | 3550000 | 41 | 100,000 | 4100000 | 0 | 150,000 | 0 |
| SC | 52 | 50,000 | 2600000 | 13 | 100,000 | 1300000 | 2 | 150,000 | 300000 |
| SE | 24 | 50,000 | 1200000 | 4 | 100,000 | 400000 | 3 | 150,000 | 450000 |
| SP | 70 | 50,000 | 3500000 | 86 | 100,000 | 8600000 | 35 | 150,000 | 5250000 |
| TO | 6 | 50,000 | 300000 | 4 | 100,000 | 400000 | 0 | 150,000 | 0 |

Continuation

| **STATE** | **CAPS AD** | **CAPS AD REF** | **CAPS AD POP** | **CAPS AD III** | **CAPS AD III REF** | **CAPS AD III POP** |
| --- | --- | --- | --- | --- | --- | --- |
| AC | 0 | 100,000 | 0 | 1 | 150,000 | 150000 |
| AL | 2 | 100,000 | 200000 | 1 | 150,000 | 150000 |
| AM | 1 | 100,000 | 100000 | 0 | 150,000 | 0 |
| AP | 1 | 100,000 | 100000 | 1 | 150,000 | 150000 |
| BA | 14 | 100,000 | 1400000 | 3 | 150,000 | 450000 |
| CE | 17 | 100,000 | 1700000 | 8 | 150,000 | 1200000 |
| DF | 4 | 100,000 | 400000 | 2 | 150,000 | 300000 |
| ES | 4 | 100,000 | 400000 | 1 | 150,000 | 150000 |
| GO | 7 | 100,000 | 700000 | 1 | 150,000 | 150000 |
| MA | 7 | 100,000 | 700000 | 0 | 150,000 | 0 |
| MG | 18 | 100,000 | 1800000 | 9 | 150,000 | 1350000 |
| MS | 3 | 100,000 | 300000 | 0 | 150,000 | 0 |
| MT | 5 | 100,000 | 500000 | 0 | 150,000 | 0 |
| PA | 6 | 100,000 | 600000 | 1 | 150,000 | 150000 |
| PB | 6 | 100,000 | 600000 | 4 | 150,000 | 600000 |
| PE | 11 | 100,000 | 1100000 | 2 | 150,000 | 300000 |
| PI | 3 | 100,000 | 300000 | 2 | 150,000 | 300000 |
| PR | 20 | 100,000 | 2000000 | 3 | 150,000 | 450000 |
| RJ | 19 | 100,000 | 1900000 | 1 | 150,000 | 150000 |
| RN | 5 | 100,000 | 500000 | 1 | 150,000 | 150000 |
| RO | 1 | 100,000 | 100000 | 0 | 150,000 | 0 |
| RR | 0 | 100,000 | 0 | 0 | 150,000 | 0 |
| RS | 22 | 100,000 | 2200000 | 4 | 150,000 | 600000 |
| SC | 10 | 100,000 | 1000000 | 0 | 150,000 | 0 |
| SE | 3 | 100,000 | 300000 | 0 | 150,000 | 0 |
| SP | 70 | 100,000 | 7000000 | 12 | 150,000 | 1800000 |
| TO | 0 | 100,000 | 0 | 2 | 150,000 | 300000 |
| CAPS: Psychosocial Care Centers; CAPS AD: Psychosocial Care Centers for Alcohol and Drugs; CAPS REF: coverage rate according to parameters established by the Ministry of Health: CAPS I: 50 (thousand inhabitants); CAPS II, CAPS AD: 100 (thousand inhabitants); CAPS III: 150 (thousand inhabitants). | | | | | | |

| **2 - 2013** | | | |
| --- | --- | --- | --- |
| **STATE** | **PRESUMED POPULATION** | **POP IBGE** | **PRESUMED COVERAGE** |
| AC | 250000 | 805014 | 31% |
| AL | 3200000 | 3247944 | 99% |
| AM | 1300000 | 3751293 | 35% |
| AP | 250000 | 744809 | 34% |
| BA | 12950000 | 14505213 | 89% |
| CE | 10150000 | 8791846 | 115% |
| DF | 1050000 | 2765696 | 38% |
| ES | 1700000 | 3736386 | 45% |
| GO | 3750000 | 6415383 | 58% |
| MA | 4500000 | 6836179 | 66% |
| MG | 14300000 | 20371328 | 70% |
| MS | 1500000 | 2589069 | 58% |
| MT | 2100000 | 3226030 | 65% |
| PA | 4500000 | 8054419 | 56% |
| PB | 4850000 | 3890494 | 125% |
| PE | 6100000 | 9189087 | 66% |
| PI | 3250000 | 3221407 | 101% |
| PR | 7300000 | 10908262 | 67% |
| RJ | 8700000 | 16616344 | 52% |
| RN | 2600000 | 3332952 | 78% |
| RO | 1250000 | 1657620 | 75% |
| RR | 500000 | 491066 | 102% |
| RS | 10450000 | 11066527 | 94% |
| SC | 5200000 | 6620186 | 79% |
| SE | 2350000 | 2173452 | 108% |
| SP | 26150000 | 43528708 | 60% |
| TO | 1000000 | 1467474 | 68% |
| BRAZIL | 141200000 | 200004188 | 71% |
