# Supplementary material for: Profile of individuals served and presumed coverage of Psychosocial Care Centers (CAPS) in Brazil: A study of the period 2013–2019
Source: PLoS One. 2024 Sep 6;19(9):e0308274. doi: 10.1371/journal.pone.0308274 (PMC11379171; doi:10.1371/journal.pone.0308274)
Supplement: S2 Table — (DOCX) [file pone.0308274.s002.docx]

| Table S2 – Calculation memory for presumed coverage of CAPS by states and Brazil in 2019. | | | | | | | | | |
| --- | --- | --- | --- | --- | --- | --- | --- | --- | --- |
| **STATE** | **CAPS I** | **CAPS I REF** | **CAPS I POP** | **CAPS II** | **CAPS II REF** | **CAPS II POP** | **CAPS III** | **CAPS III REF** | **CAPS III POP** |
| AC | 5 | 50,000 | 250000 | 1 | 100,000 | 100000 | 0 | 150,000 | 0 |
| AL | 54 | 50,000 | 2700000 | 7 | 100,000 | 700000 | 0 | 150,000 | 0 |
| AM | 15 | 50,000 | 750000 | 4 | 100,000 | 400000 | 2 | 150,000 | 300000 |
| AP | 2 | 50,000 | 100000 | 0 | 100,000 | 0 | 0 | 150,000 | 0 |
| BA | 181 | 50,000 | 9050000 | 35 | 100,000 | 3500000 | 3 | 150,000 | 450000 |
| CE | 68 | 50,000 | 3400000 | 30 | 100,000 | 3000000 | 4 | 150,000 | 600000 |
| DF | 1 | 50,000 | 50000 | 3 | 100,000 | 300000 | 0 | 150,000 | 0 |
| ES | 12 | 50,000 | 600000 | 8 | 100,000 | 800000 | 1 | 150,000 | 150000 |
| GO | 45 | 50,000 | 2250000 | 16 | 100,000 | 1600000 | 1 | 150,000 | 150000 |
| MA | 49 | 50,000 | 2450000 | 17 | 100,000 | 1700000 | 4 | 150,000 | 600000 |
| MG | 167 | 50,000 | 8350000 | 61 | 100,000 | 6100000 | 20 | 150,000 | 3000000 |
| MS | 14 | 50,000 | 700000 | 5 | 100,000 | 500000 | 3 | 150,000 | 450000 |
| MT | 31 | 50,000 | 1550000 | 3 | 100,000 | 300000 | 0 | 150,000 | 0 |
| PA | 52 | 50,000 | 2600000 | 18 | 100,000 | 1800000 | 1 | 150,000 | 150000 |
| PB | 64 | 50,000 | 3200000 | 4 | 100,000 | 400000 | 3 | 150,000 | 450000 |
| PE | 68 | 50,000 | 3400000 | 25 | 100,000 | 2500000 | 3 | 150,000 | 450000 |
| PI | 43 | 50,000 | 2150000 | 10 | 100,000 | 1000000 | 1 | 150,000 | 150000 |
| PR | 66 | 50,000 | 3300000 | 26 | 100,000 | 2600000 | 6 | 150,000 | 900000 |
| RJ | 47 | 50,000 | 2350000 | 50 | 100,000 | 5000000 | 4 | 150,000 | 600000 |
| RN | 19 | 50,000 | 950000 | 11 | 100,000 | 1100000 | 1 | 150,000 | 150000 |
| RO | 14 | 50,000 | 700000 | 3 | 100,000 | 300000 | 0 | 150,000 | 0 |
| RR | 7 | 50,000 | 350000 | 1 | 100,000 | 100000 | 1 | 150,000 | 150000 |
| RS | 80 | 50,000 | 4000000 | 43 | 100,000 | 4300000 | 1 | 150,000 | 150000 |
| SC | 63 | 50,000 | 3150000 | 15 | 100,000 | 1500000 | 2 | 150,000 | 300000 |
| SE | 30 | 50,000 | 1500000 | 4 | 100,000 | 400000 | 3 | 150,000 | 450000 |
| SP | 106 | 50,000 | 5300000 | 106 | 100,000 | 10600000 | 40 | 150,000 | 6000000 |
| TO | 10 | 50,000 | 500000 | 2 | 100,000 | 200000 | 0 | 150,000 | 0 |

Continuation

| **STATE** | **CAPS AD** | **CAPS AD REF** | **CAPS AD POP** | **CAPS AD III** | **CAPS AD III REF** | **CAPS AD III POP** | **CAPS AD IV** | **CAPS IV REF** | **CAPS IV POP** |
| --- | --- | --- | --- | --- | --- | --- | --- | --- | --- |
| AC | 1 | 100,000 | 100000 | 0 | 150,000 | 0 | 0 | 500,000 | 0 |
| AL | 2 | 100,000 | 200000 | 1 | 150,000 | 150000 | 0 | 500,000 | 0 |
| AM | 1 | 100,000 | 100000 | 1 | 150,000 | 150000 | 0 | 500,000 | 0 |
| AP | 1 | 100,000 | 100000 | 1 | 150,000 | 150000 | 0 | 500,000 | 0 |
| BA | 16 | 100,000 | 1600000 | 5 | 150,000 | 750000 | 0 | 500,000 | 0 |
| CE | 21 | 100,000 | 2100000 | 5 | 150,000 | 750000 | 0 | 500,000 | 0 |
| DF | 4 | 100,000 | 400000 | 4 | 150,000 | 600000 | 0 | 500,000 | 0 |
| ES | 4 | 100,000 | 400000 | 1 | 150,000 | 150000 | 0 | 500,000 | 0 |
| GO | 8 | 100,000 | 800000 | 2 | 150,000 | 300000 | 0 | 500,000 | 0 |
| MA | 7 | 100,000 | 700000 | 0 | 150,000 | 0 | 0 | 500,000 | 0 |
| MG | 39 | 100,000 | 3900000 | 23 | 150,000 | 3450000 | 1 | 500,000 | 500000 |
| MS | 3 | 100,000 | 300000 | 0 | 150,000 | 0 | 1 | 500,000 | 500000 |
| MT | 5 | 100,000 | 500000 | 0 | 150,000 | 0 | 0 | 500,000 | 0 |
| PA | 6 | 100,000 | 600000 | 1 | 150,000 | 150000 | 0 | 500,000 | 0 |
| PB | 5 | 100,000 | 500000 | 9 | 150,000 | 1350000 | 0 | 500,000 | 0 |
| PE | 12 | 100,000 | 1200000 | 7 | 150,000 | 1050000 | 0 | 500,000 | 0 |
| PI | 5 | 100,000 | 500000 | 2 | 150,000 | 300000 | 0 | 500,000 | 0 |
| PR | 24 | 100,000 | 2400000 | 10 | 150,000 | 1500000 | 0 | 500,000 | 0 |
| RJ | 24 | 100,000 | 2400000 | 6 | 150,000 | 900000 | 0 | 500,000 | 0 |
| RN | 7 | 100,000 | 700000 | 2 | 150,000 | 300000 | 0 | 500,000 | 0 |
| RO | 1 | 100,000 | 100000 | 0 | 150,000 | 0 | 0 | 500,000 | 0 |
| RR | 0 | 100,000 | 0 | 1 | 150,000 | 150000 | 0 | 500,000 | 0 |
| RS | 30 | 100,000 | 3000000 | 13 | 150,000 | 1950000 | 1 | 500,000 | 500000 |
| SC | 13 | 100,000 | 1300000 | 2 | 150,000 | 300000 | 0 | 500,000 | 0 |
| SE | 3 | 100,000 | 300000 | 2 | 150,000 | 300000 | 0 | 500,000 | 0 |
| SP | 78 | 100,000 | 7800000 | 19 | 150,000 | 2850000 | 0 | 500,000 | 0 |
| TO | 1 | 100,000 | 100000 | 4 | 150,000 | 600000 | 0 | 500,000 | 0 |
| CAPS: Psychosocial Care Centers; CAPS AD: Psychosocial Care Centers for Alcohol and Drugs; CAPS REF: coverage rate according to parameters established by the Ministry of Health: CAPS I: 50 (thousand inhabitants); CAPS II, CAPS AD: 100 (thousand inhabitants); CAPS III: 150 (thousand inhabitants); CAPS IV: 500 (thousand inhabitants). | | | | | | | | | |

|  | | | |
| --- | --- | --- | --- |
| **STATE** | **PRESUMED POPULATION** | **POP IBGE** | **PRESUMED COVERAGE** |
| AC | 450000 | 881935 | 51% |
| AL | 3750000 | 3337357 | 112% |
| AM | 1700000 | 4144597 | 41% |
| AP | 350000 | 845731 | 41% |
| BA | 15350000 | 14873064 | 103% |
| CE | 9850000 | 9132078 | 108% |
| DF | 1350000 | 3015268 | 45% |
| ES | 2100000 | 4018650 | 52% |
| GO | 5100000 | 7018354 | 73% |
| MA | 5450000 | 7075181 | 77% |
| MG | 25300000 | 21168791 | 120% |
| MS | 2450000 | 2778986 | 88% |
| MT | 2350000 | 3484466 | 67% |
| PA | 5300000 | 8602865 | 62% |
| PB | 5900000 | 4018127 | 147% |
| PE | 8600000 | 9557071 | 90% |
| PI | 4100000 | 3273227 | 125% |
| PR | 10700000 | 11433957 | 94% |
| RJ | 11250000 | 17264943 | 65% |
| RN | 3200000 | 3506853 | 91% |
| RO | 1100000 | 1777225 | 62% |
| RR | 750000 | 605761 | 124% |
| RS | 13900000 | 11377239 | 122% |
| SC | 6550000 | 7164788 | 91% |
| SE | 2950000 | 2298696 | 128% |
| SP | 32550000 | 45919049 | 71% |
| TO | 1400000 | 1572866 | 89% |
| BRASIL | 183800000 | 210147125 | 87% |

IBGE: Instituto Brasileiro de Geografia e Estatística

IBGE POP: resident population by state acoording to the national survey of residents per household.
